# CYP-mediated permethrin resistance in *Aedes aegypti* and evidence for *trans*-regulation

**DOI:** 10.1371/journal.pntd.0006933

**Published:** 2018-11-19

**Authors:** Letícia B. Smith, Rakshit Tyagi, Shinji Kasai, Jeffrey G. Scott

**Affiliations:** 1 Department of Entomology, Comstock Hall, Cornell University, Ithaca, New York, United States of America; 2 Department of Medical Entomology, National Institute of Infectious Diseases, Toyama, Shinjukuku, Tokyo, Japan; Liverpool School of Tropical Medicine, UNITED KINGDOM

## Abstract

*Aedes aegypti* poses a serious risk to human health due to its wide global distribution, high vector competence for several arboviruses, frequent human biting, and ability to thrive in urban environments. Pyrethroid insecticides remain the primary means of controlling adult *A*. *aegypti* populations during disease outbreaks. As a result of decades of use, pyrethroid resistance is a global problem. Cytochrome P450 monooxygenase (CYP)-mediated detoxification is one of the primary mechanisms of pyrethroid resistance. However, the specific CYP(s) responsible for resistance have not been unequivocally determined. We introgressed the resistance alleles from the resistant *A*. *aegypti* strain, Singapore (SP), into the genetic background of the susceptible ROCK strain. The resulting strain (CKR) was congenic to ROCK. Our primary goal was to determine which *CYP*s in SP are linked to resistance. To do this, we first determined which *CYP*s overexpressed in SP are also overexpressed in CKR, with the assumption that only the CYPs linked to resistance will be overexpressed in CKR relative to ROCK. Next, we determined whether any of the overexpressed *CYP*s were genetically linked to resistance (*cis*-regulated) or not (*trans*-regulated). We found that *CYP6BB2*, *CYP6Z8*, CYP9M5 and CYP9M6 were overexpressed in SP as well as in CKR. Based on the genomic sequences and polymorphisms of five single copy CYPs (CYP4C50, 6BB2, 6F2, 6F3 and 6Z8) in each strain, none of these genes were linked to resistance, except for *CYP6BB2*, which was partially linked to the resistance locus. Hence, overexpression of these four *CYP*s is due to a *trans*-regulatory factor(s). Knowledge on the specific CYPs and their regulators involved in resistance is critical for resistance management strategies because it aids in the development of new control chemicals, provides information on potential environmental modulators of resistance, and allows for the detection of resistance markers before resistance becomes fixed in the population.

## Author summary

Cytochrome P450 monooxygenases (CYPs) are one of the most important mechanism of insecticide resistance in mosquitoes. These CYP enzymes break down insecticides into non-toxic forms that can be readily excreted. An increase of CYP-mediated detoxification is commonly found in pyrethroid resistant *Aedes aegypti*, however the link between specific *CYPs* and the resistance loci have not been clearly established for this species. In this study, we measured the expression levels of nine candidate *CYPs* in two strains highly resistant to permethrin: SP, a field collected strain that was selected with permethrin for high levels of resistance and CKR, a strain that contains the resistance mechanisms from SP, but that is congenic (i.e. has the genetic background) to the insecticide susceptible strain, ROCK. We found seven overexpressed *CYPs* in SP and four in CKR, confirming their involvement in resistance. Next, we sequenced the *CYP* genes (with the exception of the duplicated ones) to determine if the genes themselves are located in the resistance locus (meaning their expression is *cis*-regulated) or not (meaning their expression is *trans*-regulated). We found no reduced polymorphisms in any of the resistant strain (SP) CYPs, suggesting that the overexpression of these CYPs (and thus CYP-mediated resistance) is *trans-*regulated.

## Introduction

*Aedes aegypti* is an important pest capable of transmitting four important human disease viruses: dengue, yellow fever, chikungunya, and Zika. Dengue, for example, causes morbidity and mortality in 141 countries across the tropical and subtropical regions of the world and is estimated to be a risk to over 50% of the world’s population [1]. Yellow fever is an endemic disease in the tropical regions of Africa and South America with a recently rising number of cases in Brazil [2,3]. Chikungunya is a disease new to the Americas as of 2013 [4] that often causes debilitating joint pains in addition to flu-like symptoms. Zika was introduced to the Americas in 2015 [5] and has generated great concerns due to its association with birth defects and Guillain-Barré syndrome [6]. Given that *A*. *aegypti* has a wide global distribution, high vector competence for several arboviruses, frequently bites humans and thrives in urban environments, it poses a serious risk to human health.

Insecticides are still the primary means to control *A*. *aegypti* in endemic areas. More specifically, pyrethroids are the most widely used class of insecticides for control of adult *A*. *aegypti* [7] in the past three decades. As a result of this continued use, pyrethroid resistance in *A*. *aegypti* is a global problem [8].

Cytochrome P450 monooxygenase (CYP)-mediated detoxification is one of the primary mechanisms of pyrethroid resistance in mosquitoes. CYPs are a large family of enzymes that metabolize both endogenous substrates and xenobiotics, such as insecticides. *A*. *aegypti* have approximately 160 *CYP* genes [9]. Several studies have directly (e.g. *in vivo* and/or *in vitro* metabolism) or indirectly (reduction in resistance with the CYP inhibitor piperonyl butoxide (PBO)) implicated CYPs as a mechanism of pyrethroid resistance in *A*. *aegypti* [8]. Elucidating the specific CYP(s) responsible for resistance is challenging because of the large number of *CYP*s and because CYP-mediated resistance can be due to overexpression of a *CYP* or to a mutation in the open reading frame of a *CYP* [10]. However, identifying these CYPs is extremely important to manage resistance because it allows us to detect resistance markers and stop insecticide use before resistance becomes fixed in the population [11]. Knowledge of the specific CYPs may also aid in the development of new insecticides and resistance inhibitors as well as allow us to better understand the influence of environmental xenobiotics in the development of insecticide resistance [12].

Most studies done to identify the *CYP*s responsible for resistance in *A*. *aegypti* have looked at changes in expression levels using unrelated strains [9,13–16]. However, when strains of different origins are used, it is not possible to determine the exact relationship between the overexpressed genes and insecticide resistance, because *CYP* expression can vary for reasons unrelated to insecticide resistance. For example, *CYP9M9* was overexpressed in the SBE strain, relative to the BORA strain [13], even though both were susceptible strains.

Increased transcription of a *CYP* resulting in resistance could be due to a change in the regulatory region of the *CYP* [17], to a change in a *CYP* regulatory protein, or an increase in the copy numbers of the *CYP* through gene amplification [18,19]. These processes of increasing *CYP* expression would give different outcomes. First, a mutation in a specific *CYP* that leads to increased expression (*cis-*regulation) would be expected to show a specific increase in only that *CYP*, and the resistance would map to that *CYP*. In contrast, if resistance is due to a mutation in a gene regulatory protein (*trans-*regulation), there could potentially be multiple *CYP*s whose expression are elevated in the resistant strain, even if only one of them is responsible for the resistance. In addition, the resistance locus would not map to the *CYP* that is overexpressed. There are now several cases where *CYP* overexpression is found in insecticide resistant strains, and the overexpression is due to *trans*-regulation of the *CYP*. Examples include *CYP6D1* in *Musca domestica* [20], *6A2* and *6A8* in *Drosophila melanogaster* [21], *6BJ*^*a/b*^, *6BJ1*, *9Z25*, and *9Z29* in *Leptinotarsa decemlineata*, and *4G7*, *4G14* and *6BQ* in *Tribolium castaneum* [22–24]. Gene amplification could also lead to increased expression of a single *CYP* or multiple *CYP* genes occurring in tandem depending on the length of the duplicated region. In this case, the resistance locus could map either to one or more of the duplicate *CYP*s.

*CYP*s as a group are rapidly evolving genes [25] and are frequently polymorphic within and between strains [26,27]. When a mutation causing resistance occurs and is under high selection pressure such that the resistance allele becomes fixed in the strain or population, the region near this mutation will have decreased amounts of polymorphisms relative to the rest of the genome [28–35]. Thus, reduced abundance of single nucleotide polymorphisms (SNPs) are useful to detect resistance loci [31–33]. The footprint of the region around the resistance locus will decrease in time, as recombination introduces back variation, but this will be a slow process. Furthermore, if a mutation in a *CYP* causes resistance, we would expect the *CYP* to have a single unique allele in the resistant strain, but be polymorphic in susceptible strains.

One of the best-characterized pyrethroid resistant strains of *A*. *aegypti* is Singapore (SP). SP developed a 1650-fold resistance to permethrin (relative to the susceptible SMK strain) after 10 generations of selection [19]. Pyrethroid resistance in SP is due to CYP-mediated detoxification and target site insensitivity (V1016G+S989P mutations in the *voltage sensitive sodium channel* [*Vssc*]). CYP-mediated resistance was unambiguously demonstrated in SP through *in vitro* and *in vivo* metabolism experiments and by PBO suppression of the resistance. Nine *CYP* genes (*CYP9M6*, *9M5*, *9M4*, *6Z8*, *6Z7*, *6F3*, *6F2*, *6BB2* and *4C50*) in SP were overexpressed >3-fold relative to the susceptible SMK strain [19]. Overexpression of four of these (*CYP6Z7*, *9M4*, *9M5* and *9M6)* was due, in part, to gene amplification. The genetic linkage of these *CYP*s, or the genetic linage of their overexpression, relative to resistance has not been investigated.

In order to understand which *CYP*s in SP map to the resistance locus, we introgressed the resistance from SP into the genetic background of the susceptible ROCK strain resulting in CYP+KDR:ROCK (CKR), a resistant strain congenic to ROCK. We then asked two questions. First, which *CYP*s overexpressed in SP are also overexpressed in CKR relative to ROCK? Second, are any of the overexpressed *CYP* genes *cis-* (map to a resistance locus) or *trans-*regulated (do not map to a resistance locus)?

## Materials and methods

### *A*. *aegypti* strains and rearing

**Fig 1:**
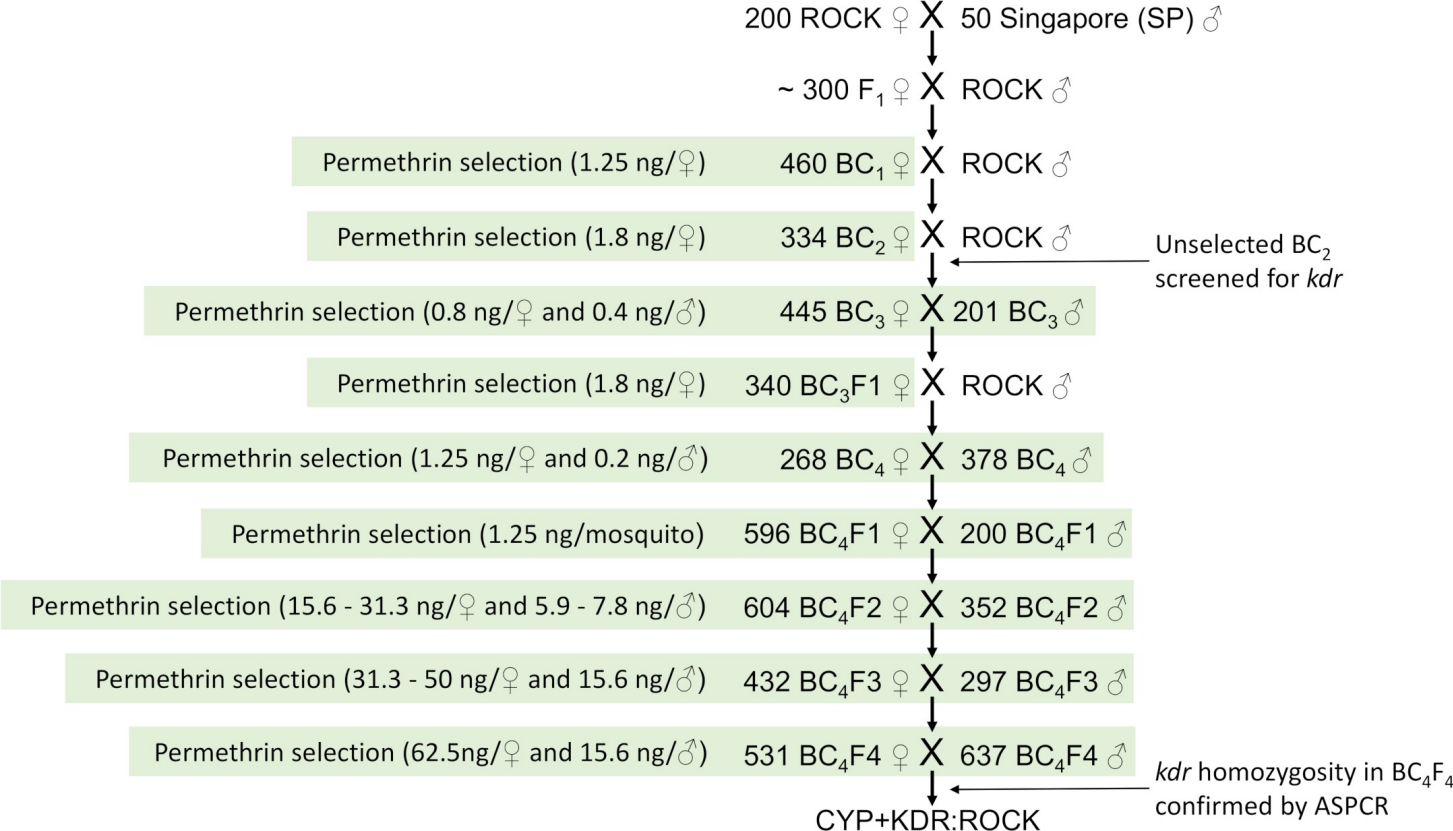
Protocol used to isolate the congenic pyrethroid resistant CKR strain. **Number of ROCK males included in each backcross varied depending on the number of females added to the cage (approximately ½ the number of females**). Permethrin selection are indicated by green shading and the doses used for each selection are shown in parenthesis.

Two parental strains of *A*. *aegypti* were used: Rockefeller (ROCK), an insecticide-susceptible strain which originated from the Caribbean [36] and has been reared without exposure to insecticides for several decades, and Singapore (SP), a pyrethroid resistant strain in which the mechanisms of resistance have been well studied [19]. SP is resistant to permethrin due to two mutations in *Vssc*, V1016G+S989P (referred to as *kdr*), and CYP-mediated detoxification, but not by hydrolases or decreased cuticular penetration [19]. A third strain, CYP+KDR:ROCK (CKR), was isolated from crossing ROCK with SP followed by four backcrosses and permethrin selections. CKR is congenic to ROCK, but resistant to pyrethroids due to CYP-mediated resistance and to *Vssc* mutations S989P+V1016G. The procedure for isolating CKR is illustrated in Fig 1. In short, unmated ROCK females were crossed *en masse* with SP males. Unmated F_1_ females were backcrossed with ROCK males and unmated BC_1_ females were selected with a permethrin dose that killed at least 60%. BC_1_ females that survived were backcrossed to ROCK males. This process was repeated for the BC_2_ and BC_3_ generations, again using doses of permethrin that gave approximately 60% mortality. To ensure that we retained all the resistance alleles, both male and unmated female BC_3_ were selected with permethrin (~60% kill) and crossed with each other prior to backcrossing to ROCK again. At BC_4_ both males and unmated females were selected with permethrin (~60% kill) and reared *en masse*. Males and unmated females from the following three generations were selected with permethrin (~60% kill) and reared *en masse*. At BC4F4, *kdr* homozygosity was confirmed by allele-specific polymerase chain reaction (ASPCR), (n = 190) following our established protocol [37]. The resultant strain was named CKR (Fig 1).

Mosquitoes were reared at 27˚C (± 1˚C) with 70–80% relative humidity, and a photoperiod of 14L:10D. Females were blood fed using membrane-covered water-jacketed glass feeders with cow blood (Owasco Meat Co., Moravia, NY). Adults were maintained on 10% sugar water in cages approximately 35 x 25 x 25 cm holding ≤ 1000 mosquitoes. Larvae (~400–600) were reared in 27.5 x 21.5 x 7.5 cm containers with 1 L distilled water and fed Cichlid Gold fish food pellets (Hikari, Hayward, CA) (ground pellets for 1st instar and medium size pellets for 2nd to 4th instars). Food pellets were given daily as needed.

### Adult bioassays

Adult bioassays were done by topical application using 3- to 7-day-old mated females. Permethrin (99.5% pure, 24.1% cis, 75.8% trans, Chem Service) and piperonyl butoxide (PBO) (90%, Sigma-Aldrich) were diluted in acetone (VWR, Radnor, PA, USA) for the bioassays. Mosquitoes were briefly anesthetized with CO_2_ and held on ice. A 0.22 μL drop of permethrin in acetone was applied to the thorax of each mosquito using a Hamilton PB-600 repeating dispenser equipped with a 10-μL syringe. Controls were treated with acetone only. At least five doses were used per bioassay with at least three giving mortality values between 0 and 100% and each containing 20 mosquitoes. Mosquitoes were given a cotton ball saturated with distilled water and held at 25˚C. A minimum of four replicates over at least two days and two cages were done per strain. Mortality was defined as mosquitoes that were ataxic after 24 h. Probit analysis [38], as adapted to personal computer use [39] using Abbott’s [40] correction for control mortality, was used to calculate the LD_50_ and the 95% confidence intervals (CI). All of the bioassay data fit a line (chi-square test). Resistance ratios (RR) were calculated by dividing the LD_50_ of the resistant strain (SP or CKR) by the LD_50_ of ROCK. Significant differences were determined by calculating the RRs for the minimum and maximum LD_50_ values based on the 95% CI. If the minimum and maximum RR values did not overlap, they were deemed significantly different. Bioassays using the synergist PBO was performed as described above, except that 2.5 μg PBO (maximum sublethal dose for the ROCK strain) was applied to each mosquito 2 h prior to permethrin application. For this, the mosquitoes were anesthetized on ice twice, once for PBO and once for permethrin application. Two controls were run: double acetone and an acetone plus PBO application.

### RT-qPCR

Ten 5–7 days old mated female mosquitos were pooled into 2 mL micro tubes (Starstedt AG & Co., Nümbrecht, Germany) containing 500 μL of TRIzol reagent (Invitrogen, Carlsbad, CA, USA) per replicate and four replicate tubes were prepared. The mosquitos were pulverized at 4.5 m/s for 20 s with an MP FastPrep 24 bead beater (MP-Biomedicals, Santa Ana, CA, USA). The RNA content was extracted following Invitrogen’s TRIzol reagent protocol. The concentration of RNA was measured using a NanoDrop 2000 spectrophotometer (Thermo Fisher Scientific Inc., Waltham, MA, USA) and then diluted to 10 μg/50μL with nuclease-free water to standardize the concentration between the tubes. DNA was removed by DNase treatment (TURBO DNA-free kit, Invitrogen) following the manufacturer’s instructions. Complete digestion of DNA was confirmed by lack of PCR amplification of the 5’ UTR of *CYP6Z7* (S1 Table) determined by visual inspection on an ethidium bromide-stained 1% agarose gel.

Complementary DNA (cDNA) was synthesized with 1 μg of total RNA per reaction using the Promega GoScript Reverse Transcription System kit (Promega, Madison, WI, USA) and random primers per the manufacturer’s instructions. The cDNA pools were then diluted 1:5 using nuclease-free water before use in real time quantitative polymerase chain reaction (RT-qPCR).

RT-qPCR plates were set up with three cDNA biological replicates and two technical replicates of each biological replicate. Two strains were compared at a time; first ROCK and SP, then ROCK and CKR. For each strain comparison, the nine *CYPs* were run along with two internal control genes, *ribosomal protein S3* (*RPS3*) and *eukaryotic translation elongation factor 1-alpha* (*EF1α*). Plates were spun down at 2100 RPM for 1 minute to ensure the liquid had reached the bottom of the wells. The reaction volume (20 μL) contained 10 μL of 2 × iQ^TM^ SYBR Green SuperMix, 7.4 μL of nuclease-free water, 0.8 μL of 10 μM of each specific primer (S1 Table), and 1 μL of first-strand cDNA template. The qPCR was performed in a CFX ConnectReal-Time PCR Detection System (Bio-Rad, Hercules, CA, USA) thermocycler with the following program: an initial denaturation and enzyme activation at 95°C for 10 min followed by 40 cycles of denaturation at 95°C for 10 s, annealing at 60°C for 10 s with a plate read, and extension at 72°C for 10 s. An automatic dissociation step cycle was added for melting curve analysis.

### Calculations

Relative quantification analysis was performed using the amplification efficiency-corrected ^ΔΔ^Ct method [41]. The change in Ct value of each strain between the target *CYP* gene and the reference gene (*RPS3* or *EF1α*) represents a ^Δ^Ct value, while the change in ^Δ^Ct value of a *CYP* between the susceptible strain (ROCK) and a resistant strain (SP or CKR) represents a ^ΔΔ^Ct, or fold-expression difference value. This is synonymous to, and will be referred to as, an R/S value in this paper. Amplification efficiency for each gene and strain was determined using LinRegPCR with a 20% exclusion of outliers from the median value, along with a manual correction of the window of linearity to fit the straight continuous set of data points in the log-linear phase of the amplification plots [42]. Data were normalized to the two endogenous controls for both strain comparisons. Multiple t-tests were conducted to determine the significance of the R/S ratios.

### Genotyping

Genomic DNA was extracted using two methods; 1) an isopropanol precipitation method from whole bodies of pooled mosquitoes, and 2) an alkali extraction from the hind legs of individual mosquitoes. The isopropanol extraction was conducted as follows: eight whole mosquitoes were placed in 2 mL tubes (Starstedt Inc., Nümbrecht, Germany) containing ten 2.3-mm diameter zirconia/silica beads (BioSpec Products, Bartlesville, OK, USA) and 400 μl Buffer A (100 mM Tris-HCl, pH 8.0, 100 mM EDTA, 100 mM NaCl, 0.5% SDS, ddH_2_O). Samples were homogenized and 800 μl of 4.3 M LiCl and 1.4 M KOAc solution was added followed by centrifugation (14,100 x g for 10 min) and collection of supernatant. Next, 570 μl isopropanol was added, mixed, centrifuged (14,100 x g for 10 min), and supernatant removed to isolate DNA pellet. Tubes containing the pellet were centrifuged (14,100 x g for 30 sec) once more with 500 μl of 70% EtOH. The supernatant was removed, then the DNA pellet was dried and resuspended in ddH_2_O. The alkali extraction method was conducted as follows: legs of individual mosquitoes were placed in individual wells of a 96-well PCR plate (BioRad, Hercules, CA, USA) containing three 2.3-mm diameter zirconia/silica beads and 10 μl 0.2 M NaOH per well. The leg samples were beaten for 1–2 min on a vortex mixer at maximum speed and then incubated for 10 min at 70˚C. Ten μL of neutralization buffer (360 mM Tris-HCl, pH 8.0 and 10 mM EDTA) and 80 μL ddH_2_O were then added to each well.

PCR was carried out using 2 μl template gDNA, 10 μl PrimeSTAR GXL Buffer (Takara Bio Inc., Shiga, Japan), 4 μl dNTP Mixture, 1 μl PrimeSTAR GXL DNA Polymerase, 2 μl forward and reverse primer mix (S1 Table, S1 Fig), 31 μl ddH_2_0 and the following thermocycler conditions: 95˚C for 3 min, 37 x (98˚C for 10 sec, 60˚C for 15 sec, 68˚C for 3 min) and 68˚C for 10 min.

### CYP sequencing and alignment

Our goal was to examine the polymorphisms in the *CYPs* that are overexpressed in the SP strain [19] to look for markers or mutations that can link the *CYPs* to resistance. To do this we sequenced *CYP* gDNA from pools of mosquitoes from the SP and ROCK strains. If polymorphisms were found in the pools, eight additional mosquitos were sequenced individually. If no polymorphism were found in the pools, this process was repeated until we had high confidence in the polymorphisms in each strain. For genes where SNPs were found, the SP sequences were compared to ROCK sequences to check for any reliable and unique SNPs in SP. We then sequenced from CKR any *CYP* with a reliable marker to examine if it was inherited from the ROCK or SP parent strain (i.e. linked to resistance or not).

ROCK and SP gDNA were sequenced and aligned for each of the single copy *CYPs* (4C50, 6BB2, 6F2, 6F3, and 6Z8) that are overexpressed in the SP strain. Four of the nine overexpressed *CYP*s found by Kasai et al. [19] could not be used to search for SNPs due to multiple gene copies; these were *CYP6Z7*, *9M4*, *9M5* and *9M6*. *CYP* sequences were determined by Sanger sequencing using PCR products treated with ExoSAP (Thermo Fisher Scientific, Waltham, MA, USA) and sequenced at the Cornell University Biotechnology Resource Center (BRC). Sequence alignments were carried out on DNASTAR’s Lasergene software, EditSeq and SeqMan Pro (Madison, WI). SNPs were searched for using the SNP Report feature in SeqMan Pro and confirmed by visually inspecting the alignments and chromatograms.

## Results

### Insecticide bioassays

CKR and SP were both resistant to permethrin (110- and 360-fold respectively) relative to ROCK (Table 1). The resistance was PBO suppressible in both CKR and SP, lowering the RR to 77- and 70-fold, respectively, confirming CYP-mediated resistance in both strains. The 3-fold difference in the resistance ratio (RR) between the CKR and SP strains suggests some minor mechanism of resistance was lost during the isolation of the CKR strain.

**Table 1:** Toxicity of permethrin +/- the CYP inhibitor PBO to the susceptible (ROCK) and resistant (SP and CKR) strains of *A*. *aegypti*.

Insecticide	Strain	LD_50_ (95% CI)	Slope (SE)	n	P^#^	RR‡
Permethrin	ROCK	1.03 (1.01–1.06)	8.1 (0.4)	400	0.23	-
Permethrin	CKR	115* (95.2–138)	2.9 (0.4)	480	0.09	112
Permethrin	SP	367*^ (302–447)	3.0 (0.5)	455	0.12	356^
PBO+permethrin	ROCK	0.29 (0.27–0.31)	6.2 (0.3)	420	0.23	-
PBO+permethrin	CKR	22.3* (22.1–22.6)	6.9 (0.1)	480	0.45	77
PBO+permethrin	SP	20.3* (20.2–20.4)	7.3 (0.1)	400	0.62	70

The average weight of ROCK, CKR and SP females used in the bioassays was 1.9, 2.5 and 1.8 mg, respectively.

#p value from chi squared test for fit of the data to the log-dose probit line.

‡RR = Resistance Ratio (LD50 of resistant strain/LD50 of susceptible strain).

*Significantly greater than ROCK.

^Significantly greater than CKR.

### CYP expression

**Fig 2:**
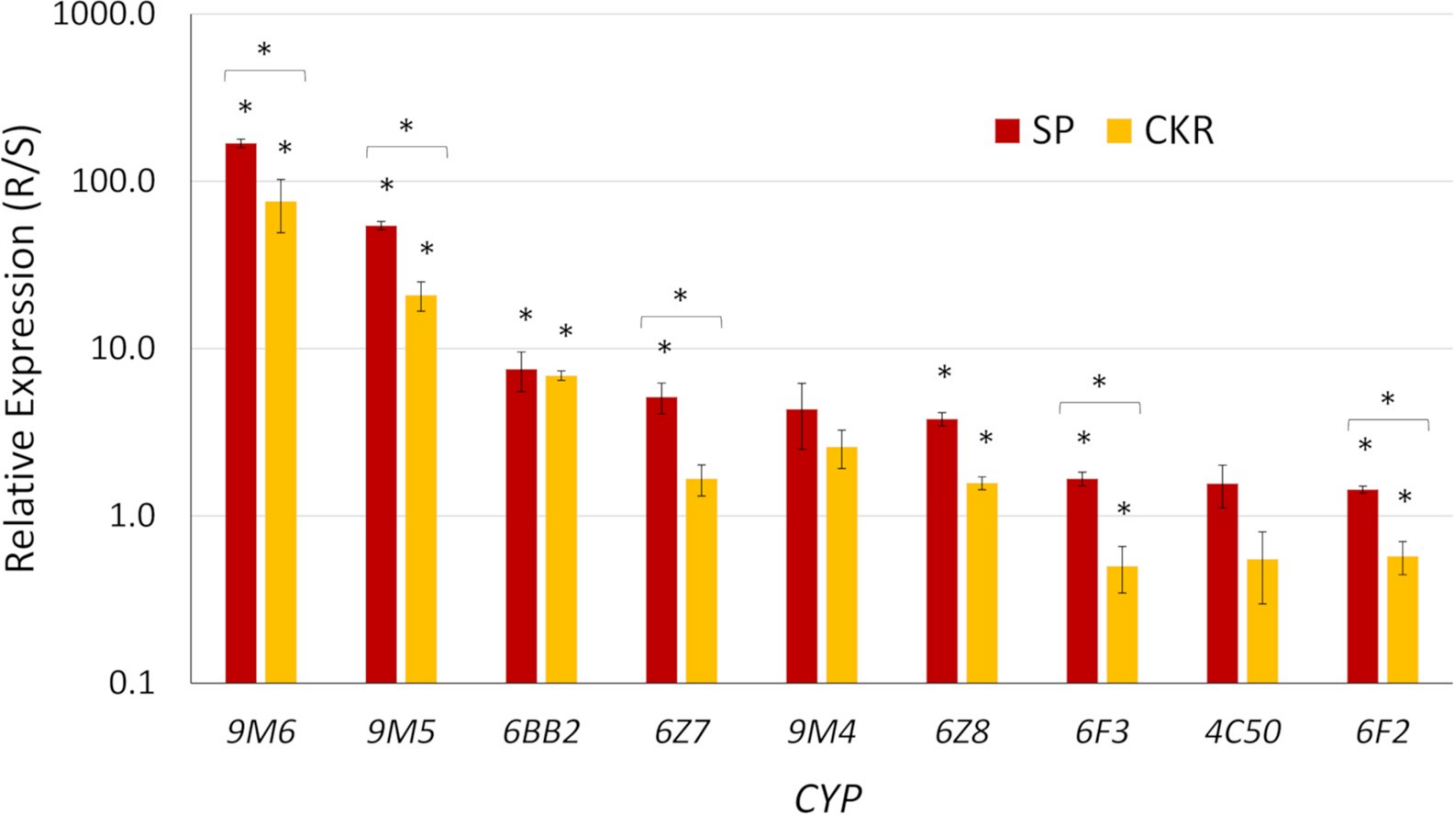
Expression of nine *CYPs* in the permethrin resistant (R) SP and CKR strains relative to the susceptible ROCK (S) strain of *A*. *aegypti*. Asterisks (*) indicate a significant difference between SP and ROCK, and CKR and ROCK or SP and CKR (P-value ≤ 0.05). Bars represent the average and standard errors from four biological replicates.

To determine if overexpression was genetically linked to permethrin resistance, *CYP* expression was quantified in both the SP and CKR strains relative to ROCK. *CYP6BB2*, *6Z8*, *9M5* and *9M6* were overexpressed in both SP and CKR indicating that their overexpression is linked to resistance. Seven *CYPs* are significantly overexpressed in the SP strain relative to ROCK: *CYP6BB2*, *6F2*, *6F3*, *6Z7*, *6Z8*, *9M5* and *9M6* (Fig 2). The level of increased expression in SP was 169 for *CYP9M6* (*p* = 7.0 x 10^−6^), 54 for *CYP9M5* (*p* = 6.6 x 10^−6^), 7.5 for *CYP6BB2* (*p* = 0.01), 5.2 for *CYP6Z7* (*p* = 0.01), 3.8 for *CYP6Z8* (*p* = 2.2 x 10^−4^), 1.7 for *CYP6F3* (*p* = 0.03), and 1.4 for *CYP6Z2* (*p* = 0.03). For CKR relative to ROCK, only *CYP6BB2*, *CYP6Z8*, *CYP9M5* and *CYP9M6* were significantly overexpressed (Fig 2). The fold- change in expression (R/S) in CKR were 76 for *CYP9M6* (*p* = 0.05), 21 for *CYP9M5* (*p* = 0.02), 6.9 for *CYP6BB2* (*p* = 2.8 x 10^−3^), and 1.5 for *CYP6Z8* (*p* = 0.03). When comparing the two resistant strains, SP had a higher level of expression of *CYP6F2* (*p* = 4.0 x 10^−3^), *6F3* (*p* = 3.0 x 10^−3^), *6Z7* (*p* = 0.01), *6Z8* (*p* = 4.8 x 10^−4^), *CYP9M5* (*p* = 1.5 x 10^−3^), and *CYP9M6* (*p* = 0.02) compared to CKR. On average, the expression in SP was about 2.4-fold greater than seen for CKR. *CYP4C50*, *6BB2* and *9M4*, and were not significantly different between SP and CKR (Fig 2).

The basal *CYP* transcription levels (^Δ^Ct values) were also investigated for each strain relative to *RPS3* and *EF1α*. Expression levels of all nine *CYP*s were readily detectable (S2 Fig, S3 Fig) and similar between both endogenous controls.

### CYP polymorphisms

Genomic DNA of five single copy *CYPs* (*4C50*, *6BB2*, *6F2*, *6F3*, *6Z8*) were sequenced from both the ROCK and SP strains (see S1 Fig for diagram of genes and approximate primer locations). GenBank accession numbers for consensus *CYP* sequences are listed in S2 Table. These *CYP*s were selected because they are overexpressed in SP, but not duplicated [19]. SP had more SNPs than ROCK in *CYP4C50*, *6F2*, *6F3 and 6Z8* (Table 2). The frequency of polymorphisms per kilobase (kb) ranged from 23–53 in SP and from 18–36 in ROCK. However, there were no strain-specific polymorphisms (i.e. neither a unique nucleotide between strains at a non-polymorphic site, nor a SNP in which both nucleotides differed between strains). This is not what would be expected for a gene at a resistance locus and leads us to conclude that the *CYP4C50*, *6F2*, *6F3 and 6Z8* genes are not linked to resistance, even though their increased expression was (see above). In contrast to these four *CYP*s, there were many less SNPs detected in *CYP6BB2*, which had 0.08 and zero SNPs per kb in ROCK and SP, respectively (Table 2). *CYP6BB2* had a unique, strain-specific synonymous polymorphism (thymine in ROCK and cytosine in SP at position 1595) that was homozygous in both strains. This allowed us to test if *CYP6BB2* was linked to resistance by sequencing this *CYP* from the CKR strain. We found seven individuals homozygous for the SP allele, 10 heterozygotes, and four homozygous for the ROCK allele in the CKR strain. This indicates a partial genetic linkage of *CYP6BB2* and the resistance locus. Based on the methods used to isolate the CKR strain, a measurement of the linkage was not possible.

**Table 2:** Total number of single nucleotide polymorphisms (SNPs) found within each strain (ROCK and SP) and proportion of unique SNPs found in only one, but not the other strain. Consensus sequences codes are available for both ROCK and SP in GenBank (S2 Table).

	ROCK			SP		
*CYP*	Total SNPs	Bases sequenced	SNP freq^a^	Total SNPs	Bases sequenced	SNP freq^a^
4C50	24	1331	18	54	1319	41
6BB2	2	2430	0.8	0	2437	0
6F2	34	1552	22	35	1552	23
6F3	55	1529	36	81	1530	53
6Z8	53	1522	35	71	1522	47

^a^Number of polymorphisms per kilobase (kb) sequenced.

## Discussion

Our bioassay results generated compelling data that we had isolated a strain (CKR) that had CYP-mediated resistance and *kdr* from the SP strain. Most of the resistance in SP was recaptured in the isolation of the CKR strain, although the 3-fold lower permethrin resistance in CKR (110-fold) compared to SP (360-fold) reveals that some resistance alleles may have been lost in the selection process. This can happen if the resistance factor is recessive and/or has a high fitness cost [43]. Interestingly, the RR value to SP is nearly 5-fold lower than that reported in Kasai et al. 2014. There are at least three possible explanations for this. First, different batches of permethrin were used and this is known to alter the expression of resistance [44]. Second, different susceptible strains were used and this can cause differences in levels of resistance reported [45]. Third, the SP strain may have lost some resistance while being maintained in the lab since it was received in 2014. Bioassays with PBO, reduced the RR to 77- and 70-fold in CKR and SP respectively, confirming the involvement of CYP-mediated resistance in both strains. The suppression of resistance with PBO was incomplete (*kdr* alone confers 40-fold resistance [37]) as is commonly seen in strains with CYP-mediated resistance [19,46]. Both CKR and SP are homozygous for the S989P+V1016G *Vssc* mutations.

Our study both validates previous work [19] and provides new information about the basis of CYP-mediated resistance in SP. Consistent with what was previously reported [19], we find elevated expression of *CYP6BB2*, *6Z7*, *6Z8*, *9M5* and *9M6* in SP. Given that we used a different susceptible strain in this study, and still found increased expression of these CYPs in SP, strengthens the hypothesis that these CYPs are involved in resistance. Further, our data provides evidence that the overexpression of four CYPs in the SP strain are genetically linked to resistance: *CYP6BB2*, *6Z8*, *9M5* and *9M6*. For *CYP9M5* and *9M6* (but not *6BB2* or *6Z8*), this is due in part to gene duplication [19].

How many different transcriptional regulation genes might be involved in insecticide resistance is an important, but unanswered question. Thus far, several different transcription factors have been implicated in insecticide resistance, including Gfi-1 in *M*. *domestica* [17,47] cap n collar C (CncC) and muscle aponeurosis fibromatosis (Maf) family transcription factors in *Tribolium castaneum* [24]. Identification of the mutation responsible for the increased expression of *CYP*s in the SP strain would expand our knowledge about this important evolutionary process and would provide a means by which the population genetics of this resistance could be studied. There is clearly some evolutionary plasticity in CYP-mediated resistance [48], but identification of the transcriptional regulatory factors, the mutations that cause *CYP* overexpression and the geographic frequency of these mutations are needed before we will start to have a satisfactory understanding of this important mechanism of resistance.

*CYP* genes are generally highly polymorphic. For example, in *Anopheles gambiae CYP*s have an average SNP frequency of 1 every 26 bp compared to the 1 every 34 bp genome average [26]. Determining the genetic diversity of *A*. *aegypti* has proven to be a challenging task due to the large genome size and high percentage of repetitive transposable elements [49]. One study found the average SNP frequency in the *A*. *aegypti* genome to be 12 per kb, however estimates of average nucleotide diversity (π) have varied greatly, ranging from about 0.001 to 0.015 [50] [51,52]. We found that the *CYP*s we studied (with the exception of *6BB2*) had a frequency of polymorphisms similar to the average reported for *An*. *gambiae* [26] with an average SNP frequency of 1 per 36 bp in ROCK and 1 per 26 bp in SP. However, *CYP6BB2* also had little variation (only two SNPs in the 2430 bp sequenced) in ROCK. The low level of polymorphism in *CYP6BB2* appears more to do with the stains we used, rather than the gene *per se*, as wild *A*. *aegypti* populations from Uganda and Senegal had 164 *CYP6BB2* SNPs, from Mexico there were 45 SNPs, and in populations from Sri Lanka there were no SNPs [50].

Overall, our results suggest that CYP-mediated resistance in SP is due to a *trans*-regulatory factor(s) that is capable of increasing the expression of multiple *CYP*s. The overexpression of four *CYPs* (*CYP6BB2*, *6Z8*, *9M5* and *9M6*) were linked to resistance. However, sequencing of the five single-copy *CYP*s that were found to be overexpressed in the SP strain, revealed that none of them showed expected signs of being at the resistance locus, except for *CYP6BB2* which showed partial linkage to a resistance locus. Given that three of the *CYPs* have multiple copies in SP precluded us from being able to evaluate their linkage to resistance. Hopefully more sequence information will become available for these amplicons in the future which would allow for testing of linkage. Based on these results and other studies [20–24,53–55], it appears that *trans*-regulation of *CYP* expression may be a common mechanism of insecticide resistance.

## Supporting information

S1 Table PCR and sequencing primers used in this study. (DOCX)

S2 Table GenBank accession numbers for the five CYPs from ROCK and SP. (DOCX)

S1 Fig Illustration of the primer locations for each of the *CYPs*. The black line indicates the exons, solid gray line indicates introns, and dashed gray lines indicates long introns that are not to scale relative to the rest of the gene.
(TIF)

S2 Fig *CYP* levels in ROCK and SP strains of *A. aegypti*. Data was normalized to ribosomal protein S3 (RPS3). Asterisks (*) indicate a significant difference between ROCK and SP (P-value ≤ 0.05). Bars represent the average and standard errors from four biological replicates.
(TIF)

S3 Fig *CYP* levels in ROCK and CKR strains of *A. aegypti*. Data was normalized to ribosomal protein S3 (RPS3). Asterisks (*) indicate a significant difference between ROCK and CKR (P-value ≤ 0.05). Bars represent the average and standard errors from four biological replicates.
(TIF)
