# Supplementary material for: CYP-mediated permethrin resistance in Aedes aegypti and evidence for trans-regulation
Source: PLoS Negl Trop Dis. 2018 Nov 19;12(11):e0006933. doi: 10.1371/journal.pntd.0006933 (PMC6277111; doi:10.1371/journal.pntd.0006933)
Supplement: S1 Table — (DOCX) [file pntd.0006933.s001.docx]

**S1 Table.** **PCR and sequencing primers used in this study.**

| Gene | Primer name | Sequence | Use |
| --- | --- | --- | --- |
| *CYP4C50* | 4C50CUF1 | GTCACGACTACGAGTTCCTG | PCR and sequencing |
| *CYP4C50* | 4C50F7 | TCAGCGATGCCTATCGATCC | PCR and sequencing |
| *CYP4C50* | 4C50CUR1 | CACTCTCGCCGACTGATC | PCR and sequencing |
| *CYP6BB2* | 6BB2F1 | GGCTTCATCCTAGAAGGATC | PCR and sequencing |
| *CYP6BB2* | 6BB2F15 | TTACGTTGCTGCGGAACTTC | PCR and sequencing |
| *CYP6BB2* | 6BB2F7 | GTGTAACTCGATTAAGAACCCG | PCR and sequencing |
| *CYP6BB2* | CYP6BB2R6 | CGATCGGTTCAATCTTCAGGTATATCC | PCR and sequencing |
| *CYP6BB2* | 6BB2R2 | CTCTACACAGTCACTCCTTC | PCR only |
| *CYP6BB2* | 6BB2R17 | GCAATTCACTGGAAGTCCTC | PCR only |
| *CYP6F2* | 6F2F1 | CCGCTTTGCGGGCTTAGTATC | PCR and sequencing |
| *CYP6F2* | 6F2F2 | GGCCTTCGCGTTTTTGAAC | PCR and sequencing |
| *CYP6F2* | 6F2F7 | GGTATGTAGTAGGTGTCTTGTCTG | PCR and sequencing |
| *CYP6F2* | 6F2R1 | CAAACATTCGAGACCCAACCATCCC | PCR and sequencing |
| *CYP6F2* | 6F2R3 | ATCACCAGTTCCGGTTCGTC | PCR and sequencing |
| *CYP6F3* | 6F3F1 | GCGCTCGCAAAGAGGTAACAACC | PCR and sequencing |
| *CYP6F3* | 6F3F7 | GGTGTGCGACTGATAACTCTC | PCR and sequencing |
| *CYP6F3* | 6F3R1 | GTCTACTCGCGACAGCTAACC | PCR and sequencing |
| *CYP6Z8* | 6Z8F1 | GTTTGTGGTCTTCACTCTTCG | PCR and sequencing |
| *CYP6Z8* | 6Z8F7 | GCGCTGCTGCATCGTTTATC | PCR and sequencing |
| *CYP6Z8* | 6Z8R2 | CCTCGATGCCAATGAAGTCC | PCR and sequencing |
| *RPS3* | aegRPF7 | AGCGTGCCAAGTCGATGAA | RT-qPCR internal control |
| *RPS3* | aegRPR8 | GTGGCCGTGTCGACGTACT | RT-qPCR internal control |
| EF1-α | EF1aF5 | CGCCCTGATTGCCAAGAGT | RT-qPCR internal control |
| EF1-α | EF1aR6 | AGCAGACCGTCCATCTCGAT | RT-qPCR internal control |
| *CYP4C50* | 4C50F5 | TACGCGTACATCCCGTTCAG | RT-qPCR |
| *CYP4C50* | 4C50R6 | CCGCCTCTATGCGGAACTTT | RT-qPCR |
| *CYP6BB2* | 6BB2F3 | AGAAAGAGCACAGCTGCGAAA | RT-qPCR |
| *CYP6BB2* | 6BB2R4 | ACTGCCGGCTGGAAGAAGTT | RT-qPCR |
| *CYP6F2* | 6F2F5 | TCCGGAGGTGAAGAGCAAAC | RT-qPCR |
| *CYP6F2* | 6F2R6 | AACTTCGACAGCAGCAGACA | RT-qPCR |
| *CYP6F3* | 6F3F1 | GGTGGCGTTTGGCATTAAGATCG | RT-qPCR |
| *CYP6F3* | 6F3R1 | CGCATGTTGTTTCGGAAGTTTG | RT-qPCR |
| *CYP6Z7* | 6Z7F5 | CAGGACTGCTGAAGTATACAGGAA | RT-qPCR |
| *CYP6Z7* | 6Z7R6 | TGAATGAAATCCTTACGCGTCACA | RT-qPCR |
| *CYP6Z8* | 6Z8F8 | CGCGAATTGGTGTCACGATG | RT-qPCR |
| *CYP6Z8* | 6Z8R9 | TGAACGCATCGTTCGGATCATTAA | RT-qPCR |
| *CYP9M4* | 9M4F5 | CCGTACACGTTGGAAGATTATGAC | RT-qPCR |
| *CYP9M4* | 9MFR6 | TTGGGATCTGGACAGCTTGTC | RT-qPCR |
| *CYP9M5* | 9M5F6 | GGAATCGTCAGCAGAAAGTGTGT | RT-qPCR |
| *CYP9M5* | 9M5R7 | CGACTGCAGTGGAATTTGAATG | RT-qPCR |
| *CYP9M6* | 9M6F88 | TCGGTGCACAATCCAAACAAC | RT-qPCR |
| *CYP9M6* | 9M6R89 | GTCGGGTACGACCAACGAAA | RT-qPCR |
| *CYP6Z7* | 6Z7_5'UTR_F | GTCGCATCAACCTGAACACGATG | gDNA contamination test |
| *CYP6Z7* | 6Z7R5 | AGCTTATCACCCACTGCCAA | gDNA contamination test |
