# Supplementary material for: CYP-mediated permethrin resistance in Aedes aegypti and evidence for trans-regulation
Source: PLoS Negl Trop Dis. 2018 Nov 19;12(11):e0006933. doi: 10.1371/journal.pntd.0006933 (PMC6277111; doi:10.1371/journal.pntd.0006933)
Supplement: S2 Table — (DOCX) [file pntd.0006933.s002.docx]

**S2 Table. GenBank accession numbers for the five CYPs from ROCK and SP.**

| **CYP** | **Strain** | **GenBank accession number** |
| --- | --- | --- |
| 4C50 | Rockefeller | MF804422 |
| 4C50 | Singapore | MH632742 |
| 6BB2 | Rockefeller | MH632736 |
| 6BB2 | Singapore | MH632737 |
| 6F2 | Rockefeller | MH632738 |
| 6F2 | Singapore | MH632739 |
| 6F3 | Rockefeller | MH632740 |
| 6F3 | Singapore | MH632741 |
| 6Z8 | Singapore | MH473729 |
| 6Z8 | Rockefeller | MH632735 |
