# Supplementary figures and images for: CYP-mediated permethrin resistance in Aedes aegypti and evidence for trans-regulation
Source: PLoS Negl Trop Dis. 2018 Nov 19;12(11):e0006933. doi: 10.1371/journal.pntd.0006933 (PMC6277111; doi:10.1371/journal.pntd.0006933)

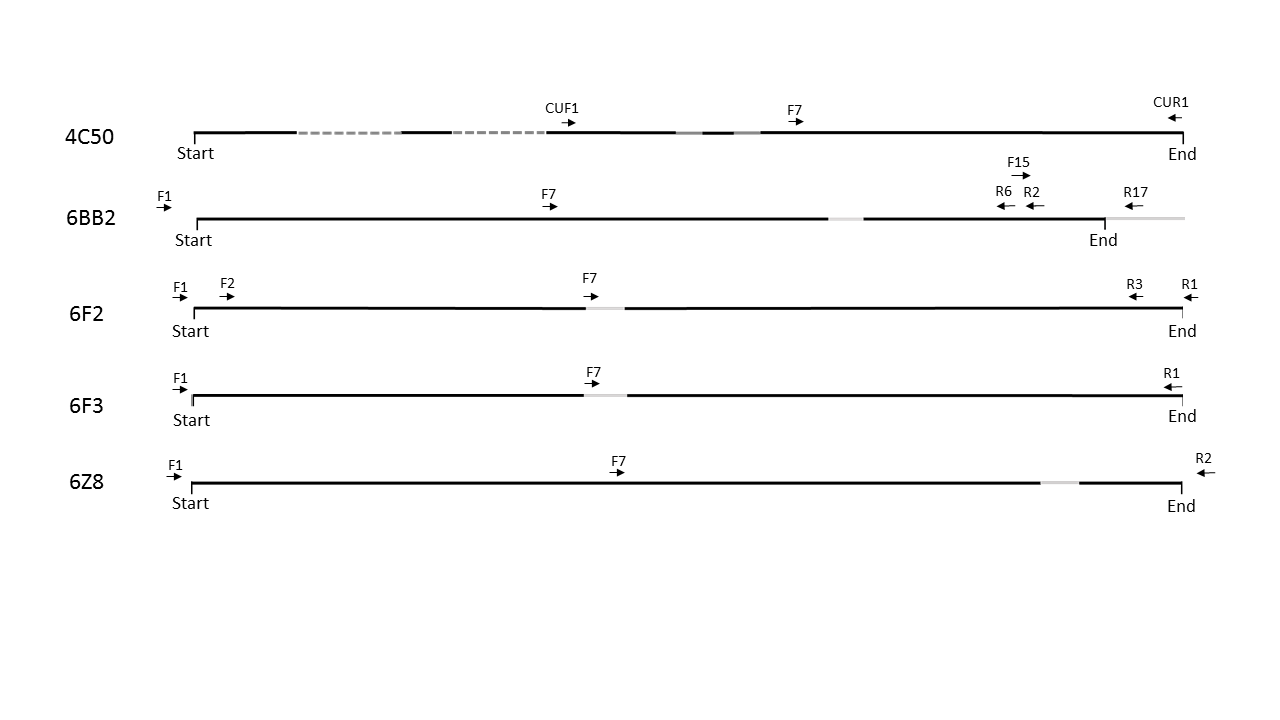

Supplement: S1 Fig — The black line indicates the exons, solid gray line indicates introns, and dashed gray lines indicates long introns that are not to scale relative to the rest of the gene. (TIF) [file pntd.0006933.s003.TIF]

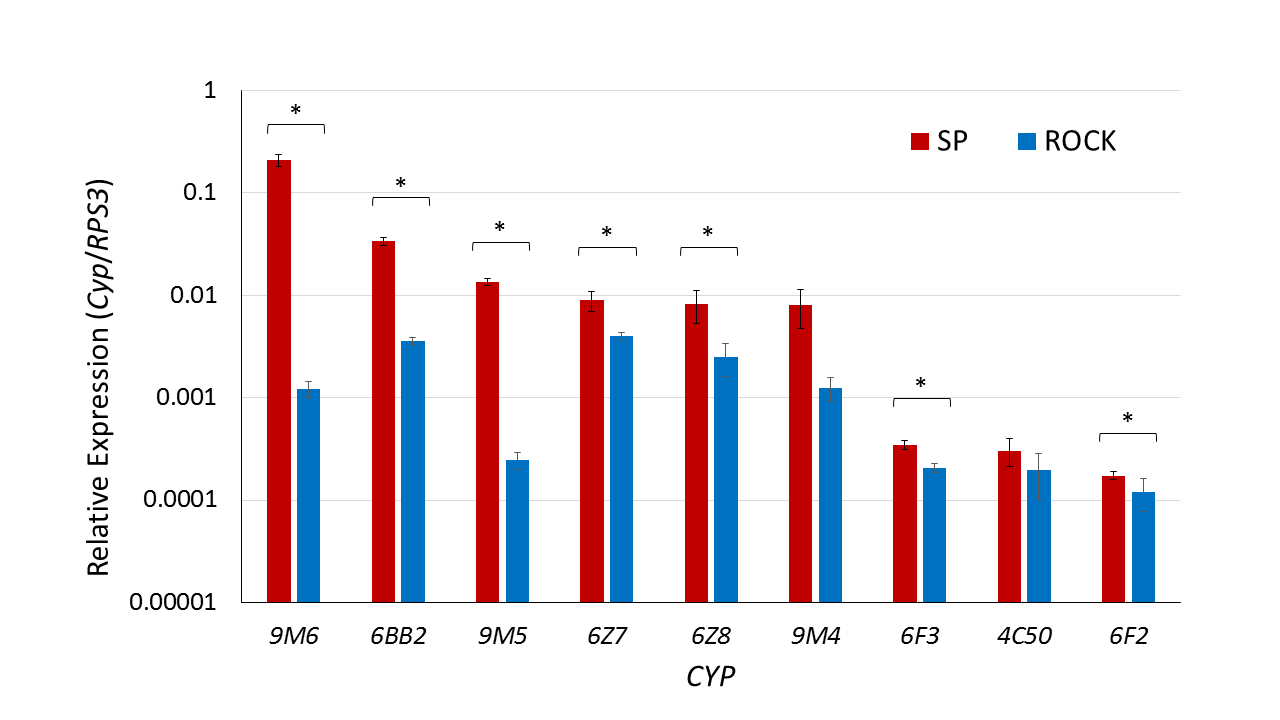

Supplement: S2 Fig — Data was normalized to ribosomal protein S3 (RPS3). Asterisks (*) indicate a significant difference between ROCK and SP (P-value ≤ 0.05). Bars represent the average and standard errors from four biological replicates. (TIF) [file pntd.0006933.s004.TIF]

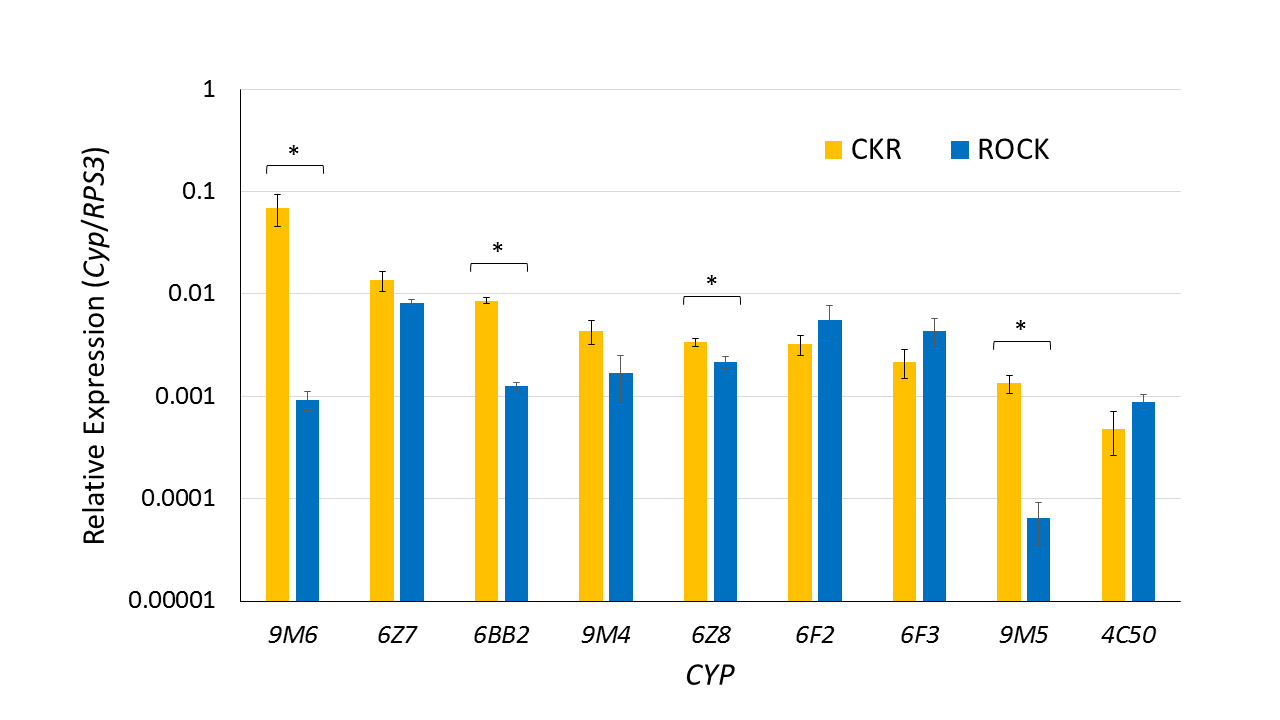

Supplement: S3 Fig — Data was normalized to ribosomal protein S3 (RPS3). Asterisks (*) indicate a significant difference between ROCK and CKR (P-value ≤ 0.05). Bars represent the average and standard errors from four biological replicates. (TIF) [file pntd.0006933.s005.TIF]
